# Patients’ and Nurses’ Perceptions of Importance of Caring Nurse–Patient Interactions: Do They Differ?

**DOI:** 10.3390/healthcare10030554

**Published:** 2022-03-16

**Authors:** Jasenka Vujanić, Štefica Mikšić, Ivana Barać, Aleksandar Včev, Robert Lovrić

**Affiliations:** 1 Faculty of Dental Medicine and Health Osijek, Josip Juraj Strossmayer University of Osijek, 31000 Osijek, Croatia; jvujanic@fdmz.hr (J.V.); smiksic@fdmz.hr (Š.M.); ibarac@fdmz.hr (I.B.); avcev@fdmz.hr (A.V.); 2 Department of Internal Medicine, University Hospital Centre Osijek, 31000 Osijek, Croatia; 3 Faculty of Medicine, Josip Juraj Strossmayer University of Osijek, 31000 Osijek, Croatia

**Keywords:** caring, clinical practice, nurses, nurse–patient interaction, health care, humanism

## Abstract

Nurse–patient interaction is a professional and therapeutic relationship created to enable nurses to assess, plan, and deliver health care aimed at meeting patients’ basic human needs. The main aim of this study was to identify distinctive characteristics and differences in perceptions between patients and nurses related to the importance of caring interactions and to examine the contribution of independent variables in explaining their perceptions. A total of 446 respondents were included in the research (291 patients and 155 registered nurses). Data were collected using the translated and standardized 70-item version of the Caring Nurse–Patient Interactions Scale (CNPI-70) version for patients and version for nurses. According to the overall CNPI-70 scale, there was a significant difference in patients’ and nurses’ perception (*p* < 0.001). Patients assessed caring nurse–patient interactions significantly higher (4.39) than nurses (4.16). Additionally, nurses assessed all subscales significantly lower than patients who assessed them high (*p* < 0.05), except for the subscales for “environment” (*p* = 0.123) and “spirituality” (*p* = 0.132). Independent variables did not contribute to an explanation of respondents’ perceptions. Providing quality physical assistance in meeting human needs through effective communication and teaching is crucial for promoting a holistic patient approach, improving psychosocial support and nurse–patient interaction, and attaining greater satisfaction with health care provided without additional financial investments.

## 1. Introduction

Nurse–patient interaction is a professional and therapeutic relationship created to enable nurses to assess, plan, and deliver health care aimed at meeting patients’ basic human needs [1]. In philosophical discussions, theories, and innovative research by renowned theoreticians, Watson, Leininger, Boykin, and Swanson, ‘caring’ is defined as the essence of nursing and is the key element of effective, high-quality nurse–patient interaction [2,3]. Person-centered care, along with safety and quality of basic care, constitute global initiatives that strengthen and reflect importance of interactions between health care professionals and their patients [4]. It is this trusting relationship that is the cornerstone of quality health care. Additionally, safe and confidential nurse–patient interaction is important for patients’ satisfaction with the care provided and treatment outcomes, as well as for nurses’ satisfaction [5]. Moreover, various authors have documented positive therapeutic effects of humane relationships on nurses in the context of improved self-esteem, well-being, sense of personal achievement, and higher job satisfaction [5,6]. The results of the latest study by Chung et al. conducted in 2021 indicate the positive correlation of the two constructs: nurses’ well-being (healthy way of living, satisfaction, joyfulness) and their competence in establishing a nurse–patient relationship. Therefore, nurses’ competences in interacting with patients significantly correlate with health-promoting lifestyle and work environment satisfaction [7]. Furthermore, other studies report the benefits of nurse–patient interactions for patients, such as improved levels of independence and autonomy, immunity, quality of life, and overall satisfaction with the care provided [8,9,10]. Furthermore, those studies describe how such caring interactions contribute to the sense of patients’ security and reduced frequency of repeated hospitalizations [8,9,10]. Although the importance of nurse–patient interactions is recognized as the basis of high-quality care, the priorities of contemporary health care are still task implementation and cost reduction [11].

Scientific literature warns about the strong influence of the biomedical model on prioritizing nursing tasks, when nurses give priority to activities ordered by doctors [12]. Although nurses feel caring and humane, they are often primarily task-oriented and provide care not considering the fact that it is extremely important for patients to experience humanity [11]. Such a lack of an individualized and humane approach in nursing is often the result of difficult working conditions and inefficient organizational culture in the clinical setting [11,12], which could consequently lead to phenomenon of rationed, unfinished, or neglected nursing care [13]. Therefore, many authors warn and express concern about the fact that nurses, in the present paradigm, deliver low-level health care with an obvious lack of compassion and humanity [8,9,11,12,13]. The problem of delivering low-quality health care significantly reduces patient comfort and impairs the quality of the health system among the population [12]. Observing the problem from patients’ perspective, Felgen et al. emphasize that health care users expect humane behavior. If they experience it, they become satisfied and loyal customers [14]. However, in the present healthcare environment, nurses value a humane approach in nursing less than their task performance and its cost. However, patients regard being treated in humane as much more important [14].

To develop relationships with patients, nurses have to base their practice on a humanistic–altruistic value system that provides patients and their families with an environment conducive to potential development. Such an approach gives nurses the opportunity to develop a holistic view of the patients they care about and attach importance to their experiences [15].

### Theoretical Framework

The relationship nurses develop with patients and their families corresponds to the humane process that Watson calls the transpersonal caring relationship [2,16]. Watson’s Theory of Human Caring is a source for studies researching nurse–patient interactions [16,17,18,19,20]. Watson defines caring as the ethical and moral ideal of health care with interpersonal and humanistic qualities [16]. In her work, she explains that the essence of nursing is not procedural parts—procedures, tasks, and interventions used in different areas—but rather the nurse–patient interaction that results in a therapeutic outcome. Therefore, its scope is not limited to nursing specialties [2,16,21]. She describes the transpersonal relationship as a relationship in which a nurse is fully committed to the patient, so that each experience becomes a real caring opportunity. Such a relationship simultaneously recognizes values and emphasizes personalities [2,16,21]. Accordingly, 10 carative factors identified in her theory focus on preparing nurses to interact with patients [2,16,21]. Carative factors represent the essence of J. Watson’s theoretical contribution. They are not linear steps towards human caring [3,16,21] (Table 1).

Carative factors described by J. Watson are well accepted in the profession because they point to the humanistic value of health care and function as structural guidelines for understanding the process of caring interpersonal relationships [3,16,21]. Watson has advanced the concept of 10 carative factors into clinical caritas processes [3,20,22]. Although the concept of clinical caritas processes is similar to the concept of 10 carative factors, clinical caritas processes have a more pronounced spiritual dimension and clearly arouse love and care [20,22]. However, the 10 original carative factors remain a timeless structural core of the theory, while caritas processes, with their fluid aspects, enable a higher level of understanding and development [2,20,22].

Finally, an outstanding contribution of Watson’s caring theory is that it combined many approaches to concepts of caring into a single source which led to designing instruments to assess the quality of caring and nurse–patient interactions [23]. The fact is that caring cannot be objectively measured and quantified [8], although special instruments can be helpful for assessing and self-assessing competence, frequency of use, and the importance of the caring nurse–patient interaction [8,24,25,26]. Numerous studies have examined caring behaviors based on Watson’s theory of human caring. They have been conducted on patients and/or nurses using a variety of instruments [6,8,18,24,25,26,27]. Regardless of the type of instrument used, they measure frequency, importance, competence, applicability, or satisfaction with caring behavior [6,8,18,24,25,26,27]. Some studies even used several measures in combination [5,18].

In 2005, Cossette developed a Nurse–Patient Interaction Scale (CNPI-70) which is considered a reliable instrument for assessing the quality of nurse–patient interaction, based on J. Watson’s Theory of caring [28]. Seventy items, organized in 10 subscales, help nurses to evaluate the caring theory in all their procedures and behaviors which exceed only being kind to patients. Due to its relevance and comprehensiveness of formulated items, the CNPI-70 scale is applicable to different groups of respondents (patients, nurses, family members, nursing students) and can measure the perception of importance, frequency of competence, and applicability of nurse–patient interaction [23,28].

Despite numerous studies on caring, a review of related literature reveals a global deficit of studies examining the specifics and possible differences in patients’ and nurses’ perceptions of importance of certain aspects of caring [20,29,30].

Therefore, this study seeks to help address this shortcoming by providing comparison results of patients’ and nurses’ perceptions of the importance of caring in clinical practice. Starting from the fact that perception and attitudes have a strong influence on the pattern of human behavior [30,31], these results could be very useful in developing and improving nurse–patient interaction, as well as humane approaches to patients during nursing care. Possible differences in patients’ and nurses’ perceptions determined in a timely manner could point to the fact that nurses do not know enough about patients’ expectations and important aspects of caring. These discrepancies in perceptions and attitudes can lead to misunderstandings and difficulties in interpersonal relationships, as well as to problems in all segments of caring. Therefore, timely determined perceptions and attitudes of patients and nurses can preventively warn of such situations and enable prompt actions.

The main aims of this study were: (a) to identify the specifics and the differences in perceptions between patients and nurses related to the importance of caring interactions; (b) to examine the contribution of sociodemographic and situational variables to explanation of patients’ and nurses’ perceptions related to the importance of caring interactions.

## 2. Materials and Methods

### 2.1. Study Design

This cross-sectional study was conducted at the Clinical Hospital Center (CHC) in the Republic of Croatia. Criteria for choosing the mentioned health institution for our study were: CHC in our study is the central and largest health institution in this region of our country. The 1400-bed institution consists of 13 clinics, 6 clinical institutes, and 7 departments. The CHC’s 3100 employees provide healthcare for around one million persons with social health insurance who gravitate to the institution. The CHC is also the largest higher education institution and the main teaching base between the two such institutions for health professionals. Furthermore, the researchers (authors of this article) are lecturers in the mentioned higher education institutions and organize clinical classes in the examined CHC. The department selection criterion is listed above. A quantitative approach to the research was applied and an anonymous survey was conducted using a closed questionnaire.

### 2.2. Respondents

In total, 446 respondents were included in the research. The first group of respondents included 291 patients hospitalized for a minimum of 48 h in the mentioned health institution (CHC) at the following departments: Department of Surgery, Department of Otorhinolaryngology, Head and Neck Surgery, Department of Gynecology and Obstetrics, Department of Internal Medicine, Department of Urology, and Department of Traumatology. The inclusive criterion (a minimum of 48 h of hospitalization) was defined based on the review of the literature on caring which suggests that brief interactions with patients limit the development of caring nurse–patient interactions based on J. Watson’s Caring theory [22,23,25,28].

The second group of respondents included 155 registered nurses, who cared for hospitalized patients in the above-mentioned departments.

In accordance with the mentioned inclusion criterion for patients, the main criterion for selecting inpatient facilities to conduct the research was the possibility of providing continuous nursing care for a minimum of 48 h [22,23,25,28]. The sample size was calculated using the online software Sample Size Calculator from Creative Research Systems [32]. The calculation of the sample size of nurses was based on the total number of nurses employed in the chosen departments and determined inclusion criteria (*n* = 209) with the initially defined values of confidence interval of 4%, confidence level of 95%, and significance level of α = 0.05. The calculation of the recommended sample size for this study was 155 nurses. The sample size of patients was also based on the total number of patients hospitalized in the chosen departments (*n* = 330) during the study period, according to the inclusion criteria, with initially defined values of confidence interval of 4%, confidence level of 95%, and significance level of α = 0.05. The calculation of the optimal sample size for this study was 213 patients.

### 2.3. Instrument

Data were collected using the translated and standardized 70-item version of the Caring Nurse–Patient Interactions Scale (CNPI-70): version for patients and version for nurses. The questionnaire, based on Watson’s Caring Theory, was developed by Cossette, Cara, Ricard, and Pepin in 2005 [28]. Seventy items of the questionnaire describe respondents’ caring behaviors and attitudes in clinical practice and can measure the importance, competence, frequency, and applicability of caring nurse–patient interactions. The questionnaire items, which reflect the carative factors described by J. Watson, are organized into 10 subscales: “humanism” (1–6), “hope” (7–13), “sensitivity” (14–19), “helping relationship” (20–26), “expression of emotions” (27–32), “problem solving” (33–38), “teaching” (39–47), “environment” (48–54), “needs” (55–64), and “spirituality” (65–70) [28]. The consent was granted by the questionnaire author, Sylvie Cossette inf. PhD, Professor, The Faculty of Nursing, University of Montreal, The Research Center, Montreal Heart Institute, Quebec, Canada.

The CNPI-70 questionnaire was translated from English into Croatian through the following steps: translation by two bilingual experts, independently; reverse translation, without any reference to the original text of the instrument; comparison of the original and translated items by another bilingual expert. It was adapted to the norms and standards of nursing care in clinical practice. The reliability of the questionnaire was tested by the Cronbach’s coefficient. The reliability of each CNPI-70 subscale ranged from 0.77 (“humanism”) to 0.90 (“problem solving”). The overall reliability of the CNPI-70 questionnaire was 0.97, indicating high reliability. A five-point Likert scale was used to measure responses about the importance of caring nurse–patient interactions. Each item was scored from 1 to 5 points (not at all = 1, a little = 2, moderately = 3, a lot = 4, extremely = 5).

### 2.4. Data Collection

Data were collected during a four-month period at the departments of the mentioned health institution. Researchers (authors of this manuscript) distributed the questionnaires to respondents, nurses, and patients, who voluntarily completed questionnaires using the pen-and-paper method. Time to complete the questionnaire was not limited. It lasted 30 min on average. Patients completed the questionnaire in the patient room at the time provided for daily rest. Completed questionnaires were collected later that same day to increase the response rate. There was a collecting box set up in every head nurse’s room of each department to collect nurses’ completed questionnaires.

### 2.5. Data Analysis

Categorical data were presented in absolute and relative frequencies. The normality of the distribution of numerical variables was assessed by the Shapiro–Wilk test. Upon validating the normality of data distribution, the transformation of predictor and criterion variables (normalization and standardization) was performed since there was significant deviation from normal distribution, but also due to predictors’ multicollinearity and various scales used to measure predictor and criterion variables. Numerical data were described by arithmetic mean and standard deviation because the variables followed normal distribution. The differences related to numerical variables between the two independent groups were examined by Student’s *t*-test. Multiple regression analysis was used to determine mutual contribution and dependence of variables. The significance level was set to Alpha = 0.05. The program used for statistical data processing was SPSS for Windows (version 22.0, IBM SPSS, Armonk, NY, USA).

### 2.6. Ethical Considerations

Prior to each data collection, the researchers thoroughly informed the respondents about the purpose of the research, ethical issues, and the details of the questionnaire. Respondents had the right to withdraw before and during the completion of the questionnaire. The anonymity of the respondents was guaranteed, thereby making it impossible to establish their identity from the answers. Only researchers had access to research data. They received the author’s consent to translate and use the CNPI-70 scale as the instrument. This study was approved by the Institutional Review Board of the CHC (IRB approval number: IRB-a: R1:8099-7.3).

## 3. Results

Out of the total respondents 291 (62.25%) were hospitalized, 140 (48.1%) female and 151 (51.9%) male patients times (Table 2). Average age was 59.77 ± 15.59 years with a range of 18–95 years. Most respondents, 146 (50.2%), lived in urban areas. They were mostly high-school graduates, 189 (64.9%). Most of them, 76 (26.1%), were hospitalized in the Department of the Internal Medicine. Most of them, 128 (44%), had been hospitalized three to five times.

The study included 155 (34.75%) registered nurses: 112 (72.3%) general nurses with high school vocational education and training (VET), and 43 (27.7%) Bachelor of Science (BSc) nurses (Table 3). Average age was 39.95 ± 12.94 years with a range of 20–64 years. There were significantly more female, 130 (83.9%), than male nurses, 25 (16%). Most of them, 96 (61.9%), lived in urban areas. Most of them, 112 (72.3%), had formal nursing education.

### 3.1. Patients’ and Nurses’ Perception of Caring Nurse–Patient Interaction

**Figure 1:**
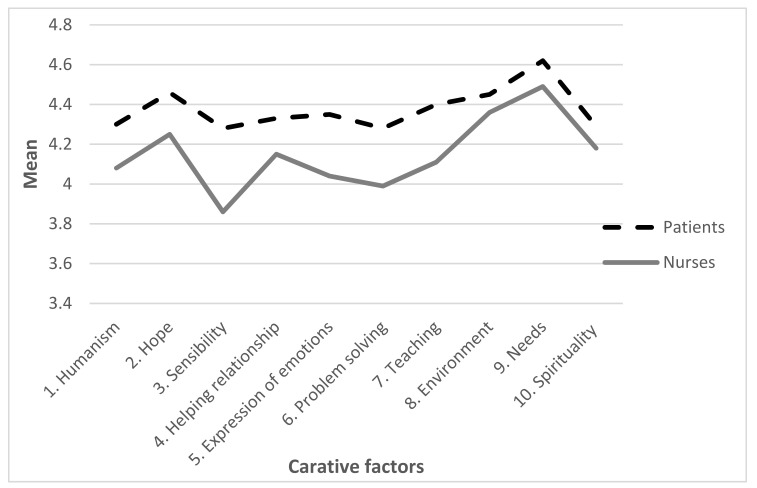
Patients’ and nurses’ perceptions of importance of Caring Nurse–Patient Interactions.

The overall mean of patient’s perception of the importance of caring nurse—patient interaction according to the CNPI-70 scale (response range 1–5) was 4.39 ± 0.48. Furthermore, the analysis of patients’ perception of specific subscales of CNPI-70 scale indicated significant differences (*p* < 0.01). Patients attached the greatest importance to subscale “needs” (4.62 ± 0.47), while subscale “problem-solving” was assessed the lowest (4.28 ± 0.74) (Figure 1 and Table 4).

Nurses’ perception according to the CNPI-70 scale was 4.17 ± 0.46 (Table 4). There were significant differences in nurses’ perception in relation to certain subscales (*p* < 0.01). The most important subscale was “needs” (4.49 ± 0.47), while the least important subscale was “sensibility” (3.86 ± 0.47) (Figure 1 and Table 4).

### 3.2. Differences in Patients’ and Nurses’ Perception of the Importance of Caring Nurse–Patient Interactions

According to the overall CNPI-70 scale, there was a significant difference in patients’ and nurses’ perception (*p* < 0.001). Patients assessed caring nurse–patient interactions significantly higher (4.39) than nurses (4.16) (Table 4). Additionally, nurses assessed all subscales significantly lower than patients who assessed them high (*p* < 0.05), with the exception of subscales “environment” (*p* = 0.123) and “spirituality” (*p* = 0.132) (Table 4).

### 3.3. Contribution of Independent Variables to Explanation of Nurses’ and Patients’ Perception of Caring Interaction

Sociodemographic and situational variables do not contribute to explanation of criteria for patients’ perceptions related to the importance of caring interactions. The final regression model—which included patients’ age, gender, level of education, place of residence (urban/rural), department, and number of hospitalizations—explains only 2.2% variance (R^2^ = 0.022, F (5.289) = 1.07; *p* = 0.379), making the model insignificant (Table 5).

Similar to patients’ results, sociodemographic and situational variables do not contribute to explanation of criteria for nurses’ perceptions related to the importance of caring interactions. The final regression model—which included nurses’ age, level of education, place of residence (urban/rural), workplace, and length of service—explains only 1.8% variance (R^2^ = 0.018, F (4.154) = 0.55; *p* = 0.738), making the model insignificant (Table 5).

## 4. Discussion

### 4.1. Patients’ Perception of the Importance of Caring Nurse–Patient Interactions

The overall mean of patients’ perception of the importance of caring nurse–patient interaction according to the CNPI-70 scale was 4.39 (response range 1–5), suggesting that patients attach significant importance to caring nurse–patient interactions.

The obtained result is in line with the results of the studies conducted in Saudi Arabia [33], the Philippines [34], and six European countries: Cyprus, the Czech Republic, Finland, Greece, Hungary, and Italy [35]. Although patients in this study attached significant importance to nurse–patient interactions, the results indicated significant differences in their perception in relation to subscales. Patients assessed the “needs” subscale the highest, which points to the fact that patients attach the greatest importance to dignified nursing care while their basic human needs are met. Other studies also emphasize the importance patients attach to basic physiological needs [35,36,37]. Merrill et al. report that patient-respondents identify the importance of meeting their physiological needs in a safe and timely manner to be an important area of nursing care [37]. This is further supported by Papastavrou et al. [35]. In the study conducted in Saudi Arabia, patients assessed the subscale “needs” as the third most important, while subscales “humanism” and “hope” were assessed higher. Therefore, the results of that study show that a humane approach and their value system are more important to patients than meeting their basic human needs [33].

In this study, patients recognized the importance of the subscale “environment”. This finding correlates with the results of the study by Lynn et al., in which patients assessed the subscale “environment” the highest [38]. Several studies indicate the nature of the clinical setting as an important determinant in building and maintaining a therapeutic relationship with patients [12,36,39]. In addition to the subscales “environment” and “hope”, the subscale “teaching” was assessed to be equally important by patients in this study. This may point to the fact that it is extremely important for patients to raise the level of their knowledge and skills, independence, and responsibility in order to preserve and improve their own health. While explaining procedure guidelines, nurses teach patients using appropriate teaching strategies, methods, and procedures, such as critical thinking and effective communication [16,27]. It should also be emphasized that other studies confirm hospitalized patients’ need for such explanations which result in reduced level of concern and increased sense of safety [7,8,9,40].

The subscales “humanism”, “sensibility”, “helping relationship”, “expression of emotions”, “problem-solving”, and “spirituality” were perceived as significantly less important by patients than the abovementioned subscales. Other studies also confirm that patients attach less importance to emotional subscales as there is a widespread belief among them that nurses play a major role in managing symptoms, but a small role in providing psychosocial care, which made them reluctant to express their psychosocial concerns [41,42]. This may indicate that patients are unaware of the role of nurses in emotional subscales. A study by Tay et al. supports this fact since it found that many oncology patients did not think that provision of psychosocial care, such as emotional resolution and counseling, fall under the scope of nursing practice [42]. Authors Song et al. state that patients’ perception of nurses’ workload and working conditions in the clinical setting, such as lack of time and hectic work, can also change their patterns of communication [43]. Patients respect nursing workload and job requirements. They feel uncomfortable thinking of themselves as an additional burden, hence expressing their physical pain only when it becomes unbearable [43]. The same is confirmed by the authors Chan et al. [41] who add that patients prioritize when to call a nurse and call them only when they feel it is important, mainly because of physiological changes and needs. They try not to disturb the nurses, so they look for a suitable time to express their concerns and feelings because they consider themselves less urgent to deal with [41].

All of the above-mentioned findings suggest that it is necessary to instruct patients on the importance of more open communication that will contribute to creating conditions for better development of nurse–patient interactions. Traditionally, patients still consider the role of the nurse to be focused solely on meeting physical needs and are unlikely to expect emotional care, although it does not mean it is less important to them, especially at a time when they are having a traumatic experience [41,42,43].

### 4.2. Nurses’ Perception of Importance of Caring Nurse–Patient Interactions

The results of this study show that the overall mean of nurses’ perceptions of the importance of caring nurse–patient interactions according to the CNPI-70 scale was 4.17 (response range 1–5), indicating that nurses attach importance to caring nurse–patient interactions. This is consistent with the results of the Philippine study where the overall mean of CNPI-70 was 4.20, which is considered excellent [34]. The results of other studies are similar [6,44].

However, although the results of this study and other relevant studies show relatively high scores of the total value according to the CNPI-70 scale, the analysis of differences in their perceptions by subscales show significant dissimilarities. Correspondingly, the mean of nurses’ perception in this study for the “needs” subscale was the highest of all. The results of the study by Vujanić et al. [30] also show that nurses in clinical practice most often apply procedures and behaviors from the subscale “needs”, which can be explained by the fact that attitudes have an important influence on behavior [30,31].

This study showed that nurses consider the subscale “sensibility”—cultivation of sensitivity to one’s self and to others, which belongs to humane caring together with the subscales “humanism” and “hope”—to be the least important. This finding is opposed to the results of the study by Youssef et al. which show the subscales “humanism, “hope”, and “sensibility” to be the most important subscales to nurses [45]. Respondents in this study described caring as a process that includes not only professional knowledge, competence, skills, and care, but also feelings and emotions [45].

Loscin also emphasizes the importance of all dimensions of caring and warns about technological progress, which on the one hand contributes to patients’ sense of security and reduces the costs in healthcare system, but on the other hand may reduce face to face relationships and so jeopardize the human element in caring [46].

### 4.3. Differences in Patients’ and Nurses’ Perceptions Related to the Importance of Caring Nurse–Patient Interactions

There were significant differences between the overall means of patients’ and nurses’ perceptions of the importance of caring nurse–patient interactions according to the CNPI-70 scale in this study. The results showed that nurses perceive the nurse–patient relationship as significantly less important than patients. These results may indicate that nurses are more aware of the concept of care and its dimensions due to their professional development and acquired knowledge. Therefore, they set higher standards when assessing caring behaviors and attitudes compared to patients who rely more on intuition [47]. Additionally, nurses’ perception of nursing care implementation and its significance might decrease over time, which is supported by other studies [47,48]. This phenomenon could be explained by inhibitory effects many professional environments have, since their management is strictly economically oriented and places importance exclusively on task performance, disregarding the nurse–patient interaction and its effects [49]. On the other hand, patients do not have professional knowledge of the concept of care and in their assessment rely more on their intuition and their sensitivity to nurse–patient interaction, as well as awareness of nurses’ presence, being there and being ready to provide care, which increases patients’ feeling of safety and the perception of importance of caring [50]. Furthermore, the results of this study differ from the study by Aupia et al. in which the overall result of the perception of caring behavior suggests that there is no significant difference between nurses and patients [29]. However, the results of other studies show that nurses consider caring behaviors more important than the patients do [34,35].

Differences in respondents’ perceptions of caring behaviors in the abovementioned studies could be explained by differences in sample sizes, processes and models of caring, as well as by differences in education, geographical area, and culture. Additionally, some of the cited authors conducted their research in multiple hospitals or used other versions of the questionnaire to assess caring behavior [29,34].

The results of this study also suggested significant differences in patients’ and nurses’ perceptions in relation to specific subscales of the CNPI-70 scale. The nurses assessed the subscales “hope”, “expression of emotions”, “problem solving”, and “teaching” as significantly lower than the patients did. This may point to the fact that it is very important to patients to feel the presence of a nurse who gives them hope and offers help, along with expressing and accepting positive and negative feelings [51]. Talk, explain, teach, and inform are the most common active verbs used when describing conversation with patients. This type of nursing help usually involves listening. It is impossible to determine the quality of listening provided by nurses [51]. However, if listening is passive, without given feedback, the interaction will not be satisfactory [51]. Patients need to know that their verbalization of concerns is heard and understood. Likewise, identifying negative feelings as a problem to be addressed requires application of appropriate types of help, such as teaching and explaining [51]. This is supported by the results of the study by Kullberg et al. which show that patients who are more actively involved in their own care during hospitalization often start a conversation on their own and turn to nurses for information about their illness and self-help, which in turn promotes communication between nurse and patient and enables partnership [52].

The subscale “problem-solving” was assessed significantly lower by nurses than by patients in this study. These results are worrying because this subscale represents solving problems by effectively implementing nursing processes which lead to improved health care since it facilitates patients’ healing process and reduces their hospital stay. This ultimately increases patient satisfaction with nursing care provided and the quality of caring interactions [53]. Shortened hospitalization correlates with a reduction in costs in the health care system, hence the application of all phases of nursing process also has an economic effect. Furthermore, nursing processes enable nurses to perform their activities with logical justification and help them to function as an autonomous and separate profession [53]. The nursing process is a scientific approach to problem solving. However, the level of application of nursing process varies among countries, although it is implemented almost all over the world and in some countries even used as a standard of nursing care [54].

The subscale “sensibility” was scored significantly higher by patients. The results of a study by Thorup [55] suggest that, for patients, it is most important to be understood by nurses. Patients expect nurses to understand their feelings and the effects their illness has on their life and wellbeing in general [55]. Sick people’s feelings of uncertainty and insecurity increase their need for attention, understanding, and respect as human beings [55]. Sensitivity to patient vulnerability seems to be important for the pursuit of ethically based practice. It requires understanding of the actual situation of the patient, seen from their perspective, which can only arise from nurses’ competence and courage to develop a quality interaction with patient [55].

Subscale “needs” was scored significantly higher by patients than by nurses. Some authors attribute such results to the impact of the biomedical model on nursing practice, as well as to the fact that nurses still consider medical interventions to be their primary task [30,41,56]. This may be the reason why both nurses and patients attach more importance to physical assistance provided by nurses. However, some authors believe that this should be taken advantage of since the focus on patients’ physical comfort can improve their psychological well-being [41,56]. Therefore, nurses could consider promoting patients’ psychosocial comfort by improving the quality of physical care through successful communication during nursing procedures and reducing or eliminating symptoms. This could be the solution for balanced caring in the given time frame and achieving health care goals [41].

In this study, there were no significant differences in nurses’ and patients’ perceptions according to the subscale “spirituality”, which is in line with the studies by other authors [57]; but is contrary to the results of the study by Delmas et al., which show that there are differences in the perception of spirituality between nurses and patients [17]. Nurses assessed the subscale “spirituality” significantly higher than patients, but both groups of respondents assessed this subscale the lowest of all [17]. Some authors are of the opinion that nurses may not have acquired enough knowledge and skills during formal education to provide this type of care. This could be the reason for the low assessment of this subscale, but not an indication that this subscale is not important to them. Patients, on the other hand, view the concept of care as trust and safety, not as spirituality, which is still culturally shaped by religious practices [57]. Furthermore, spirituality is the only subscale delivered not only by nurses, but also by priests, clergy, and volunteers associated with religious organizations [58]. The presence of a priest can affect the relationship with health professionals, especially if care is not patient-centered and patients feel that spirituality is outside the scope of nursing care [58]. The results of this study demonstrate that independent variables do not contribute to explanation of patients’ perception related to the importance of caring interactions, which is in accordance with the other study [59]. Some studies state that caring interactions have higher importance for female patients [29], elderly patients [29,35,60], and patients with lower level of education [29,35]. Additionally, independent variables in this study do not contribute to explanation of nurses’ perception related to the importance of caring interactions. This is to a greater or lesser extent in accordance with the results of other studies which state that there are no significant differences in nurses’ perceptions according to age [29], gender [29], level of education [29,61], and length of clinical experience [29]. In contrast, the results of some studies indicate that higher significance was given to caring interactions by older nurses [24,61,62], nurses of a higher level of education [1,10,24], and nurses with more clinical experience [1,10,24,61,62]. Hence, for both respondent groups in this study—independently of their sociodemographic or situational factors—nurse–patient caring interactions are valued highly, which indicates the overall value of this construct.

### 4.4. Further Research and Practice

We believe that further research should focus on nursing students to identify their perceptions of importance of caring interactions. Furthermore, additional longitudinal studies should be conducted using different research methods (quantitative, qualitative, and mixed) to identify the impact of the application of carative factors on patient satisfaction.

### 4.5. Limitations of the Study

The study had certain limitations. Firstly, the research was conducted in one clinical hospital center. We believe that it should be extended to other clinical centers in order to gain a better insight into the differences in patients’ and nurses’ perceptions of the importance of caring interactions. Secondly, the results of this study—directly related to specific items of the CNPI-70 scale—were compared with the results of only a few available studies that used the CNPI-70 scale to assess the importance of nurse–patient interactions. Finally, the third limitation of the present study is the extremely high reliability of CNPI-70 scale, which is usually the characteristic of unidimensional factors. This implies the possibility of bias in respondents’ answers or other potential biases. Therefore, it is necessary to consider these limitations when understanding and interpreting the results of this study, and when planning and conducting similar studies using CNPI-70 scale. It is important to mention that the CNPI-70 scale is a very congruent tool that is applicable to different groups of respondents (patients, nurses, family members, nursing students) and can measure the perception related to importance, frequency of competence, and applicability of nurse–patient interaction.

### 4.6. Implications for Nursing

This study contributes to improving knowledge related to caring behavior and better understanding of nurse–patient interactions on a global level. The contribution of this research is in the identified subscales of importance and their differences in caring nurse–patient interactions that allow self-correction and quality development of relationships with patients based on a scientific approach and new evidence-based practice. Better quality of nurse–patient interactions can significantly improve working environment, ensure a higher level of nurse and patient satisfaction, and a higher level of patient safety. Finally, it will have a positive effect on health care, one that goes beyond routine tasks, and does not require substantial financial investments.

## 5. Conclusions

Although the importance of caring nurse–patient interactions was assessed highly by patients and nurses, significant differences were found between their perceptions. Nurses attach significantly less importance to nurse–patient interactions than patients. Significant differences in the perception of importance also exist in all subscales, except in the subscales for “environment” and “spirituality”.

The conducted research on patients’ and nurses’ perceptions of the importance of nurse–patient interactions provided several valuable indicators for these two groups of respondents and suggested the need for self-correction aimed at improving effective communication, personal contact with patients, teaching, and information provision to patients and their family members. Providing quality physical assistance in meeting human needs through effective communication and teaching is crucial for promoting a holistic patient approach, improving psychosocial support and nurse–patient interaction, and greater satisfaction with health care provided without additional financial investments.

## Figures and Tables

**Table 1:** Carative factors, their descriptions.

Carative Factors (F1–F10)	Description
F1—Humanism	Formation of a humanistic-altruistic system of values. Humanistic-altruistic feelings and acts provide the basis of human caring and promote the best professional care, and as such, constitute the first and most basic factor for science and ethic of caring.
F2—Hope	Instillation of faith-hope. In this carative factor (CF), patients’ beliefs are encouraged, honored, and respected as significant influences in promoting and maintaining health.
F3—Sensibility	Cultivation of sensitivity to one’s self and to others. Nurses who recognize and use their sensitivity promote self-development and self-actualization and are able to encourage the same growth in others. Without this factor, nursing care would fall.
F4—Helping relationship	Development of a helping-trusting, human caring relationship. The human caring relationship is transpersonal. In that it connotes a special kind of relationship: a connection with the other person, a high regard for the whole person, and their being-in-the-world.
F5—Expression of emotions	Promotion and acceptance of the expression of positive and negative feelings. The caring relationship can move to a deeper, more honest, and authentic level if the nurse allows for this CF.
F6—Problem solving	Systematic use of a creative problem-solving caring process. This process involves full use of self and all of one’s faculties, knowledge, instincts, intuition, aesthetics, technology, skills, empirics, ethics, personal, and even spiritual knowing.
F7—Teaching	Promotion of transpersonal teaching–learning. This CF makes explicit that learning is more than just receiving information and data. It involves a caring relationship as context for any teaching learning.
F8—Environment	Provision for a supportive, protective and/or corrective mental, physical, societal, and spiritual environment. The areas that involve this factor are: comfort, privacy, safety, cleanliness, and aesthetic surroundings.
F9—Needs	Assistance with the gratification of human needs. All needs are equally important and must be valued and responded to for caring-healing.
F10—Spirituality	Allowance for existential–phenomenological–spiritual forces. This CF allows for spiritual filled meanings and unknowns to emerge open to infinite possibilities for miracles.

**Table 2:** Sociodemographic characteristics of patients (*n* = 291).

Respondents Characteristics		Number (%)
Gender	male	151 (51.9)
	female	140 (48.1)
Age (years)	18–40	38 (13.1)
	41–60	87 (29.9)
	61–90	166 (57.0)
Place of Residence	urban	146 (50.2)
	rural	145 (49.8)
Level of Education	elementary school	70 (24.1)
	high school	189 (64.9)
	higher education	32 (11.0)
Department	Traumatology and Orthopedics	56 (19.2)
	Surgery	22 (7.6)
	Gynecologic Oncology	30 (10.3)
	Urology	23 (7.9)
	Otorhinolaryngology	18 (6.2)
	Oncology	66 (22.7)
	Internal Clinic—Cardiology	76 (26.1)
Number of hospitalizations	1–2	123 (42.3)
	3–5	128 (44.0)
	6 and more	40 (13.7)

**Table 3:** Sociodemographic characteristics of nurses (*n* = 155).

Respondents Characteristics		Number (%)
Gender	Male	25 (16.1)
	Female	130 (83.9)
Age (years)	18–25	28 (18.1)
	26–40	54 (34.8)
	>40	73 (47.1)
Place of Residence	Urban	96 (61.9)
	Rural	59 (38.1)
Level of Education	General Nurses (VET)	112 (72.3)
	BSc nurses	43 (27.7)
Length of Service (years)	≤5	38 (24.5)
	6–20	45 (29.0)
	>21	72 (46.5)
Workplace	Traumatology and Orthopedics	40 (25.8)
	Surgery	34 (21.9)
	Gynecologic Oncology	15 (9.7)
	Urology	7 (4.5)
	Otorhinolaryngology	11 (7.1)
	Oncology	21 (13.5)
	Internal Clinic—Cardiology	27 (17.4)

**Table 4:** Patients’ and nurses’ perceptions of importance of Caring Nurse–Patient Interactions.

Carative Factors (F1–F10)	Patient Perception		Nurses’ Perception		*p* *
	Average	SD	Average	SD	
F1—Humanism	4.30	0.65	4.08	0.56	<0.001
F2—Hope	4.46	0.59	4.25	0.51	<0.001
F3—Sensibility	4.28	0.70	3.86	0.64	<0.01
F4—Helping relationship	4.33	0.65	4.15	0.58	<0.01
F5—Expression of emotions	4.35	0.66	4.04	0.62	<0.001
F6—Problem solving	4.28	0.74	3.99	0.63	<0.001
F7—Teaching	4.40	0.62	4.11	0.55	<0.001
F8—Environment	4.45	0.61	4.36	0.55	0.132
F9—Needs	4.62	0.47	4.49	0.46	<0.01
F10—Spirituality	4.29	0.57	4.18	0.57	0.109
In total (CNPI-70)	4.39	0.48	4.17	0.46	<0.001

* Independent sample *t*-test.

**Table 5:** Multiple regression analysis of individual contribution of predictor variables related to nurses’ and patients’ perception of the importance of caring interaction.

Patients	**Predictors**	**Criteria (Respondents’ Perceptions)**	
		* β	***p***
	age	0.046	0.446
	gender	0.000	0.994
	level of education	−0.049	0.415
	place of residence (urban/rural)	0.109	0.067
	department	0.070	0.252
	number of hospitalizations	−0.056	0.353
		Regression model ^†^ R = 0.149, ^‡^ R^2^ = 0.022, ^§^ R^2^corr. = 0.002, ||F (_5.289_) = 1.07, *p* = 0.379	
Nurses	age	0.128	0.759
	level of education	0.084	0.308
	place of residence (urban/rural)	−0.002	0.998
	workplace (department)	−0.015	0.855
	length of service	−0.101	0.807
		Regression model ^†^ R = 0.135, ^‡^ R^2^ = 0.018, ^§^ R^2^kor = −0.015, ||F (_4.154_) = 0.55, *p* = 0.738	

* β = regression coefficient; ^†^ R = coefficient of multiple correlation; ^‡^ R^2^ = coefficient of determination; ^§^ R^2^corr. = adjusted R^2^; ||F = F ratio.

## Data Availability

All data generated analyzed during the current study are available from the corresponding author on reasonable request.
